# The role of short-chain fatty acids in spinal cord injury: A systematic review of human and animal evidence

**DOI:** 10.1080/10790268.2025.2607831

**Published:** 2026-02-19

**Authors:** Sina Rinderknecht, Alessandro Bertolo, Ezra Valido, Stevan Stojic, Samford Wong, Gary J. Farkas, Priya Iyer, Ivana Jaric, Jivko Stoyanov, Marija Glisic

**Affiliations:** a Institute of Social and Preventive Medicine, University of Bern, Bern, Switzerland; b Swiss Paraplegic Research, Nottwil, Switzerland; c National Spinal Injuries Centre, Stoke Mandeville Hospital, Aylesbury, UK; d School of Health & Psychological Sciences, University of London, London, UK; e Royal Buckinghamshire Hospital, Aylesbury, UK; f Department of Physical Medicine and Rehabilitation and The Miami Project to Cure Paralysis, University of Miami Miller School of Medicine, Miami, Florida, USA; g The University of Sydney, Nutrition and Dietetics Group, Susan Wakil School of Nursing and Midwifery, The Charles Perkins Centre, Sydney, Australia; h Institute of Laboratory Animal Sciences, University of Zurich, Zurich, Switzerland

**Keywords:** Spinal cord injury, Short-chain fatty acids, Microbiome, Rehabilitation, Neurological recovery, Systematic review

## Abstract

**Context:**

Spinal cord injury (SCI) disrupts gut microbiota composition, resulting in dysbiosis that can worsen neuroinflammation and impede post-injury recovery. Short-chain fatty acids (SCFA), metabolites produced by the gut microbiome with anti-inflammatory properties, offer a promising avenue for improving recovery and rehabilitation outcomes.

**Objective:**

We aimed to compile a summary of the human and animal evidence on the potential benefits of SCFA or SCFA  – producing bacteria in individuals with SCI.

**Methods:**

Three databases (EMBASE, Medline (Ovid) and Web of Science) were searched from inception until 19 October 2023. No language restrictions were applied. Title and abstract screening, data extraction and risk of bias assessments were done independently by two reviewers.

**Results:**

A total of 2492 studies were retrieved, 69 full-text studies were reviewed, and 13 studies were included (11 animal and 2 human). Human studies, which involved participants with chronic SCI, linked gut dysbiosis (a proxy for low SCFA production) and human metabolic profiles, suggesting a potential role for microbiome-targeted interventions even in later stages of injury. Evidence from animal studies, predominantly in acute and sub-acute models of SCI, consistently associated SCFA interventions with improved motor function, reduced tissue damage and favorable changes in inflammatory and oxidative stress markers. Fecal microbiota transplantation and probiotics improved motor function and reduced lesion size in animal models. Gut microbiome modulations through treatments such as melatonin, moxibustion, and intermittent fasting was correlated with improved motor outcomes and increased abundance of SCFA-producing bacteria.

**Conclusions:**

This review highlights the potential of targeting the gut microbiota and SCFAs as therapeutic strategies for SCI recovery. However, despite promising results in animal models, human evidence remains limited.

## Introduction

The gut microbiota represents a complex ecosystem of bacteria, fungi, and viruses residing in the digestive tract. It acts as a dynamic and essential component of human physiological processes, influencing health both positively and negatively by producing metabolic byproducts and signaling molecules that impact the function of various organs (1, 2). Among these, short-chain fatty acids (SCFA) such as acetate, propionate, and butyrate have garnered significant attention for their potential roles in various diseases (1, 2). SCFA are primarily produced through the microbial fermentation of dietary fibres, including resistant starch, simple sugars, and polysaccharides, by gut bacteria (2). These metabolites exhibit anti-inflammatory and immunoregulatory properties and play pivotal roles in neuro-immuno-endocrine regulation (3). Notably, SCFAs influence microglial function, promote neurogenesis, regulate lipid, cholesterol, and glucose metabolism, support immune responses, and contribute to the maintenance of gut barrier integrity (2, 4).

Individuals with spinal cord injury (SCI) exhibit significant alterations in gut microbiota composition, leading to dysbiosis, which is characterized by reduced microbial diversity and disrupted structural composition (5, 6). Dysbiosis in people with SCI is marked by a decline in beneficial bacteria and an increase in pathogenic, pro-inflammatory species (7). This microbial imbalance is often due to factors characteristic for individuals with SCI, such as antibiotic use, chronic stress, and gastrointestinal dysfunction (8, 9). Studies have highlighted a significant reduction in key SCFA-producing bacteria, such as *Faecalibacterium* and *Coprococcus*, in individuals with SCI (10). This is particularly pronounced in those with motor-complete injuries, where there is a marked decrease in *Bacteroides*, *Faecalibacterium*, and members of the *Lachnospiraceae* family, indicating a loss of health-promoting microorganisms and a corresponding decline in SCFA bioavailability (10) Conversely, an increased abundance of potentially pathogenic bacteria, including *Enterococcus*, *Streptococcus*, and *Klebsiella*, has been observed (10). These microbial shifts are associated with heightened risks of urinary tract infections, bacteremia, and increased mortality in critically ill patients (11, 12).

Recent preclinical evidence has highlighted the potential benefits of short-chain fatty acids SCFA in promoting neurological recovery following neurotrauma in animals. SCFA have been shown to support recovery in experimental stroke models, both functionally and morphologically (13–15). In animal models of traumatic brain injury (TBI), SCFA treatment improved white matter connectivity, supported the energy metabolism of neurons and glia, preserved normal behavioral patterns, and maintained a healthy gut microbiota. Additionally, SCFA were shown to reduce the expression of inflammatory genes in microglia while enhancing the expression of heat shock proteins, which are activated in response to cellular stress and help protect against its harmful effects (6–18). Furthermore, SCFA administration has been reported to reduce necrotic cavity size and the infiltration of inflammatory cells in affected spinal tissue in animal models of SCI (16, 17) SCFAs also promote intestinal homeostasis by increasing villus height and mucosal thickness in the ileum (16, 17).

Therefore, there is an increasing body of evidence from both human and animal studies suggesting that the association between SCI, gut microbiota, and SCFA could offer a promising approach for improving recovery and health outcomes in individuals with SCI. This potential is largely attributed to the anti-inflammatory and antioxidative properties of SCFA. However, it is important to note that research in this area is still evolving, and gaps in our current understanding remain.

To this end, we aimed to contribute to the broadened understanding of this field by reviewing the current available literature. We performed a systematic review of research studies in both animals and humans, exploring the role of SCFA or SCFA-producing bacteria in SCI. Our goal is to provide insights into this promising avenue for improving outcomes in individuals with SCI and to identify areas that warrant further investigation.

## Methods

### Data sources and search strategy

This review followed the guidelines published by Muka *et al.* (18) and reporting adhered to the Preferred Reporting Items for Systematic Reviews and Meta-Analyses (PRISMA) (19). The search strategy, detailed in Appendix 1, included terms related to SCFA and the gastrointestinal microbiome, such as bacterial load, dysbiosis, and microbiota. These were combined with terms specific to SCI, including spinal cord ischemia, paraplegia, and quadriplegia. Three electronic databases were searched (Embase, Medline (Ovid), and Web of Science) without date and language restrictions. The date of the last search was19 October 2023, and titles and abstracts were exported into EndNote (EndNote x9; Clarivate, Philadelphia, PA, USA).

### Study selection and eligibility criteria

Studies were if they met the following criteria: (1) were conducted in humans with SCI or in animal models of SCI; (2) were designed as cohort, case–control, cross-sectional studies or randomized controlled trial, pre–post studies, or experimental pre-clinical studies; (3) assessed the association between endogenous levels of SCFA in the blood or stool or between SCFA-producing bacteria and any recovery or health-related outcome (*e.g.* neurological classification of injury, motor and sensory function, inflammation markers, etc.); (4) explored the effectiveness of SCFA supplements or other supplements aimed at enhancing SCFA production in relation to any recovery or health-related outcome. Letters to the editor, reviews, commentaries, case studies, conference abstracts, and non-peer-reviewed studies were not eligible for inclusion. Two reviewers independently screened the titles and abstracts for inclusion and assessed eligibility based on their full text. References cited in the final included studies were screened to identify additional relevant articles. Discrepancies were resolved by consensus, and a third reviewer was consulted if necessary.

### Data extraction

Two reviewers independently extracted data using a predesigned table that included the name of the lead author and publication year, study location, study design, intervention duration/study follow-up, exposure/intervention and control type, personal characteristics such as age, sex, health status/comorbidities, clinical characteristics of SCI, and changes in serum/stool SCFA or SCFA-producing bacteria on health outcomes.

### Assessing the risk of bias

The National Heart, Lung, and Blood Institute (NHLBI) Quality Assessment Tool assessed risk of bias in observational and intervention studies involving humans (20, 21). This tool focuses on the internal validity of the study and includes items to evaluate potential flaws in study methods or implementations. In particular, the tool assesses potential sources of bias (*e.g.* patient selection), confounding, study power, the strength of causality in the association between interventions and outcomes and other factors. To evaluate risk of bias, two reviewers independently went through a checklist and selected “yes”, “no” or “cannot determine (CD)/not reported (NR)/ not applicable (NA)” in response to each item on the tool.

For animal studies the SYRCLE risk of bias (RoB) tool, an adapted version of the Cochrane RoB tool, was used for risk assessment (22). The SYRCLE’s RoB tool has been adjusted to address aspects of bias that play a specific role in animal intervention studies. The tool consists of 10 questions related to selection bias, performance bias, detection bias, attrition bias, reporting bias, and other biases. Two reviewers independently chose between the possible responses: (1) “yes” which indicated a low risk of bias; (2) “no” which indicated a high risk of bias; and (3) “unclear” which indicated that risk of bias could not be assessed due to insufficient information provided within the study. Discrepancies in risk assessment were resolved by consensus and consultation with a third reviewer if needed.

### Studies synthesis

Due to significant clinical heterogeneity among the included studies  – reflected in differences in study populations, designs, and health outcomes  – a meta-analysis was not appropriate. Instead, we present a narrative synthesis of the evidence, distinguishing between human and animal studies, as well as categorizing them based on exposures or interventions used across the studies including SCFA, gut microbiota dysbiosis and other interventions.

## Results

### Study characteristics

**Figure 1.**
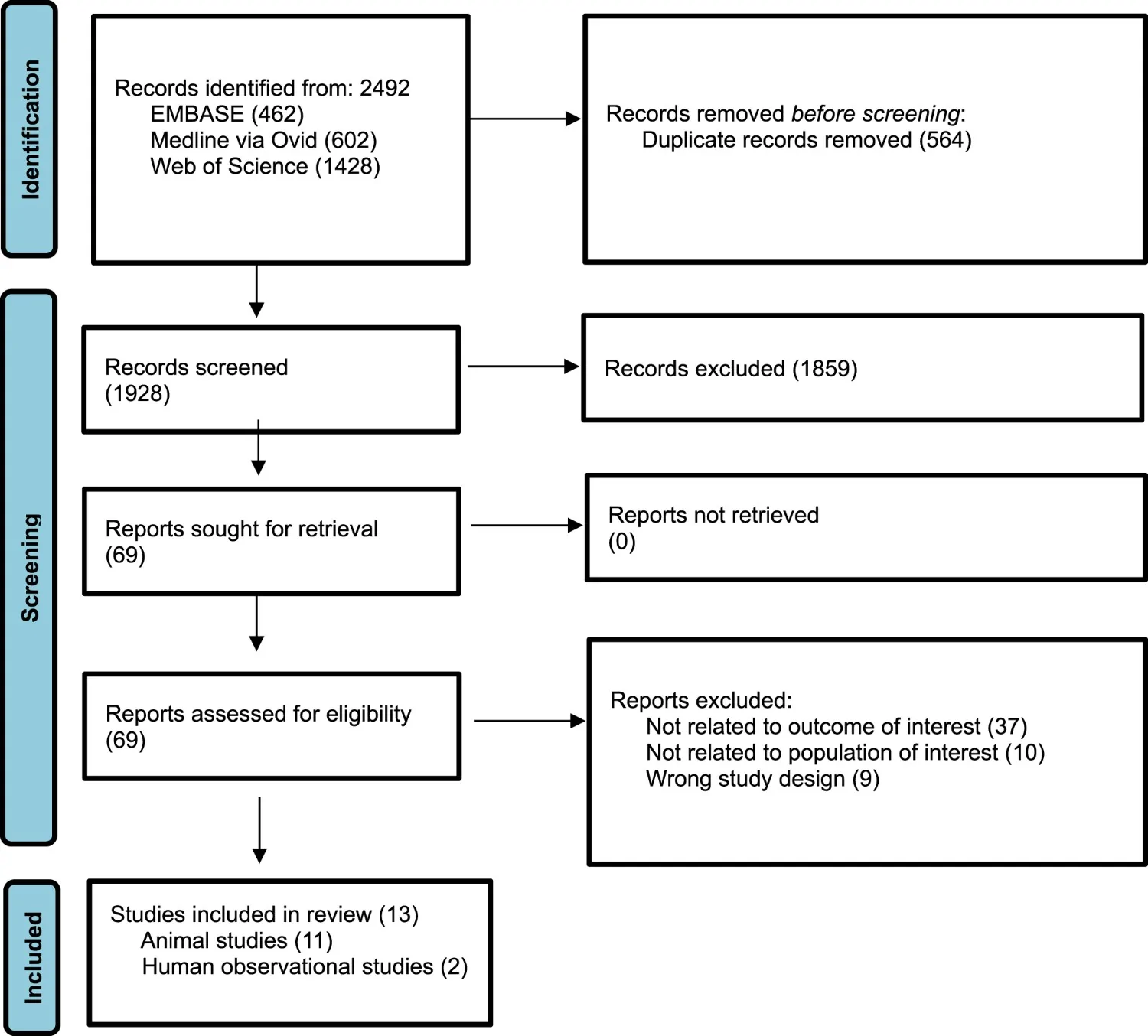
Flowchart of included studies: PRISMA flow diagram for the studies included in the current systematic review resulting from searches of databases, screening, reasons for exclusions and description of included studies.

In the initial search, we identified 2492 studies. From these, 1928 unique titles and abstracts were screened, and 69 full-text studies were retrieved, of which 13 met the inclusion criteria (Figure 1). Among the included studies, 11 were animal studies, and two were human observational studies (Table 1 and Supplemental Table 1). Five studies examined the effectiveness of SCFA or interventions aimed at increasing SCFA production and recovery post-SCI (23–27). Further, three studies (two of which were conducted in humans) associated gut dysbiosis (28–30) with recovery post-injury, two studies linked microbiome changes to dietary interventions (31, 32), and three studies connected microbiome changes resulting from melatonin, moxibustion (MOX), and recombinant interleukin 13 (rIL-13) interventions to various outcomes (33–35). Motor function was the most commonly reported outcome, assessed in nine animal studies (81.8%), followed by histological changes in spinal tissues (n = 5), spinal tissue inflammation and oxidative stress markers (n = 4), and changes in gut microbiome and SCFA-producing bacteria (n = 4). The most commonly used animal models in the research were female mice (n = 7, 63.6%), including four studies with C57BL/6 mice with contusion injuries, one with wild-type BALB/cJRj, one with C47BL76 mice, and one with Sprague–Dawley rats. Male models included two studies with CD1 mice, one with Sprague – Dawley rats, and one with male Long Evans rats.

**Table 1.** Characteristics of included studies.

Lead author, publication year	Study design	Study population	Injury characteristics/SCI model	Exposure/Intervention	Comparison groups	Outcome measures
Kong *et al.* 2023	Case-control study	11 individuals with SCI and 10 healthy controls	Chronic, cervical and thoracic SCI	Not applicable	SCI vs. healthy controls	Microbiome and serum metabolite profiles
Zhang *et al.* 2019	Case-control study	43 men with SCI and 23 healthy controls	Chronic, cervical and thoracic SCI	Not applicable	SCI vs. healthy men	Microbiome changes and gastrointestinal symptoms
Filippone *et al.* 2020	Interventional study	Male CD1 mice, n = 160	Acute Th6-7 spinal extradural compression injury	Sodium Propionate	Vehicle and sham	Motor function, inflammatory and oxidative stress markers
Lanza *et al.* 2019	Interventional study	Male CD1 mice, n = 120	Acute Th6-7 spinal extradural compression injury	Sodium Butyrate	Vehicle and sham	Motor function, inflammatory and oxidative stress markers
Liu *et al.* 2023	Interventional study	Female Spraque-Dawleay Rats, n = 65	Acute Th10 spinal contusion injury	Acetate, Propionate and Butyrate Sodium	Water	Motor function, inflammatory and oxidative stress markers
Ning He *et al.* 2022	Interventional study	Female C57BL/6 mice, n = NR	Acute Th10 spinal hemisection injury	Resveratrol and fecal microbiota transplantation	Sham	Motor function
Jing, Y *et al.* 2021	Interventional study	Adult female C57BL/6N mice, n = 120	Acute Th10 spinal contusion Injury	Fecal microbiota transplantation	Vehicle	Motor function and metabolic parameters
Kigerl *et al.* 2016	Interventional study	Female C57BL/6 mice, n = NR	Acute Th9 spinal contusion injury	Probiotic after antibiotic-induced dysbiosis	Vehicle	Motor function and metabolic parameters
Wang *et al.* 2023	Interventional study	Male Sprague-Dawley rats, n = 72	Acute C5 spinal injury through temporary aneurysm clamp	Intermittent fasting	Every other day fasting in healthy, SCI and sham	Microbiome changes
Smith *et al.* 2022	Interventional study	Male Long Evans rats, n = NR	Acute Th10 spinal contusion jury	High-fat diet and low-fat diet	Sham	Microbiome changes
Hamad *et al.* 2023	Interventional study	Female wild-type (WT) BALB/cJRj mice, n = 20	Acute Th8 spinal hemisection injury	Recombinant interleukin-13	Vehicle	Motor function and microbiome changes
Z. Zhang *et al.* 2022	Interventional study	Female C47BL76 mice, n = 32	Acute Th10 spinal contusion injury	Moxibustion	Vehicle	Motor function and microbiome changes
Jing *et al.* 2019	Interventional study	Female C57BL/6 mice, n = 64	Acute Th10 contusion injury	Melatonin treatment	Vehicle and sham	Motor function, microbiome changes and metabolic parameters

In human studies, chronic spinal cord injury was typically defined as an injury persisting for more than 12 months. In contrast, in animal models, the term “acute SCI” generally refers to the immediate phase following injury, during which early interventions are tested.

### Methodological quality of included studies

Tables 2 and 3 present the key methodological limitations in human and animal studies, respectively. In human studies, these limitations are primarily associated with the lack of power calculations and insufficient descriptions of the control selection process. In contrast, the situation in animal studies is more severe, as none of the included studies met all the criteria established by SYRCLE’s tool for assessing methodological quality. All animal studies were classified as having either some risk or an unclear risk of bias. Additionally, due to selective reporting, a major issue affecting the reproducibility of preclinical animal research, data on selection bias (such as baseline characteristics and allocation concealment), performance bias (including random housing and blinding of study personnel), and detection bias (randomized outcome assessment) were largely unavailable across most animal studies.

**Table 2.** Risk of bias assesment of human studies.

Lead Author, Publication Year	Study Design	Risk of Bias Signalling questions (listed below)											
		1	2	3	4	5	6	7	8	9	10	11	12
Kong G, 2023	Case-control	Yes	Yes	No	NR	Yes	Yes	NR	No	Yes	Yes	NA	Yes
Zhang C, 2019	Case-control	Yes	Yes	No	Yes	Yes	Yes	NR	No	Yes	Yes	NA	No

Signalling questions used to assess risk of bias of cohort/cross-sectional studies: 1. Was the research question or objective in this paper clearly stated and appropriate? 2. Was the study population clearly specified and defined? 3. Did the authors include a sample size justification? 4. Were controls selected or recruited from the same or similar population that gave rise to the cases (including the same timeframe)? 5. Were the definitions, inclusion and exclusion criteria, algorithms or processes used to identify or select cases and controls valid, reliable, and implemented consistently across all study participants? 6. Were the cases clearly defined and differentiated from controls? 7. If less than 100 percent of eligible cases and/or controls were selected for the study, were the cases and/or controls randomly selected from those eligible? 8. Was there use of concurrent controls? 9. Were the investigators able to confirm that the exposure/risk occurred prior to the development of the condition or event that defined a participant as a case? 10. Were the measures of exposure/risk clearly defined, valid, reliable, and implemented consistently (including the same time period) across all study participants? 11. Were the assessors of exposure/risk blinded to the case or control status of participants? 12. Were key potential confounding variables measured and adjusted statistically in the analyses? If matching was used, did the investigators account for matching during study analysis?

**Table 3.** Risk of bias assessment for animal studies.

Studies		Jing *et al*. (35)	Kigerl *et al*. (28)	Jing, Y *et al*. (36)	Wang *et al*. (32)	Smith *et al*. (31)	Hamad *et al*. (34)	Lanza *et al*. (27)	Liu *et al*. (26)	Zhang *et al*. (30)	Ning He *et al*, 2022	Filippone *et al*. (23)
Selection bias	Sequence generation (1)	Yes	Yes	Unclear	No	Unclear	Unclear	No	Yes	Unclear	No	Yes
	Baseline characteristics (2)	No	Yes	Yes	Unclear	Yes	Yes	Yes	Yes	Yes	Yes	Yes
	Allocation concealment (3)	Unclear	Unclear	Unclear	Yes	Yes	Unclear	No	No	Unclear	No	Unclear
Performance bias	Random housing (4)	Unclear	No	No	No	Unclear	No	No	No	No	No	Yes
	Blinding of study personnel (5)	Yes	No	No	No	No	No	No	No	No	No	No
Detection bias	Random outcome assessment (6)	No	Unclear	No	No	Yes	Unclear	No	No	No	No	No
	Blinding of outcome assessors (7)	No	Yes	Yes	Yes	No	Unclear	Yes	Yes	Unclear	No	No
Attrition bias	Incomplete outcome data (8)	Unclear	Yes	Yes	Yes	Unclear	No	Yes	Unclear	Yes	No	Yes
Reporting bias	Selective outcome reporting (9)	Yes	Yes	Yes	Yes	Yes	Yes	Yes	Yes	Yes	Yes	Yes
Other	Other sources of bias (10)	Yes	Yes	Yes	Yes	Yes	Yes	Yes	Yes	Yes	Yes	Yes

“yes” indicates a low risk of bias; “no” indicates a high risk of bias; “unclear” indicates that risk of bias could not be assessed due to insufficient information provided within the study.

### SCFA

Five studies, all conducted in animals, examined the effectiveness of SCFA or interventions aimed at increasing SCFA production and recovery post-injury (23–27). Two studies used male CD1 mice with Th6-Th7 injuries and applied sodium butyrate (SB) (27) and sodium propionate (SP) (23). One study applied an SCFA mixture in female Sprague–Dawley rats with Th10 injuries (26). Two studies used female C57BL/6 mice with Th10 injuries and looked at fecal microbiota transplantation (FMT) only or resveratrol-treatment combined with FMT (24, 36).

SB treatment improved motor function, ameliorated histopathological changes, and significantly decreased levels of pro-inflammatory and oxidative stress mediators in SCI mice compared to vehicle-treated controls (27). A dose-dependent effect was observed, with higher doses of SB (30 and 100 mg/kg) resulting in faster and more substantial motor recovery than the lower dose (10 mg/kg); similar dose-dependent improvements were noted in inflammatory and oxidative stress markers (27). Comparable effects were seen in another study with SP treatment, which significantly improved motor function in SCI mice starting as early as the third day post-injury compared to vehicle-treated controls (23). SP also provided substantial protection against SCI-induced tissue damage. At 30 and 100 mg/kg, SP treatment significantly reduced peroxidase activity indicating lower neutrophil infiltration and decreased malondialdehyde (MDA) levels more effectively than the 10 mg/kg dose (23). Furthermore, SP significantly reduced Cyclooxygenase-2 (COX-2) and inducible nitric oxide synthase (iNOS) expression, while increasing levels of the NF-kappa-B inhibitor alpha (IκB-α). In addition, activation of astrocytes and microglia in the spinal cord was markedly reduced in mice receiving SP at 30 or 100 mg/kg compared to the untreated SCI group (23).

In another study orally administered SCFA (acetate sodium, propionate sodium and butyrate sodium) enhanced hind limb motor function in rats starting 14 days post-injury [26). The intervention also reduced inflammatory cell infiltration and spinal cord necrosis, elevated the anti-inflammatory Interleukin 10 (IL-10) levels and decreased pro-inflammatory Interleukin-17 (IL-17) and γδ T-cell proliferation both *in vitro* and in the spinal cord. Additionally, SCFA treatment improved intestinal barrier function by increasing villus height, mucosal thickness, and the abundance of Treg cells in the lamina propria of the small intestine (26).

Two other studies indirectly associated SCFA with functional recovery after injury by exploring the link between SCFA-producing bacteria and motor function recovery (24, 36). Resveratrol, a natural polyphenol, improved intestinal dysbiosis and increased the relative abundance of Erysipelotrichales (order), Lactobacillales (order), and Dubosiella (genus) and decreased that of Clostridiales (order) and Lachnospiraceae (family member of Clostridiales) (24). Resveratrol treatment significantly increased the content of butyrate in serum and feces. Following fecal microbiota transplantation (FMT) from resveratrol-treated mice to recipient SCI mice, the concentration of butyrate increased in feces and was negatively correlated with microglial activation (24). FMT from age-matched healthy donors significantly improved motor function, as indicated by higher Basso Mouse Scale (BMS) scores (36). Moreover, FMT-treated mice exhibited a larger amplitude in motor-evoked potentials measured at the gastrocnemius muscle, suggesting enhanced neuronal survival and axonal regeneration, though no differences in motor-evoked potential latencies were observed (36). FMT promoted neuronal survival and axonal regeneration after traumatic SCI. FMT resulted in greater weight gain and restored average respiratory exchange quotient values in SCI mice. Gastrointestinal transit was accelerated, and intestinal barrier integrity was maintained in the FMT group (36). Additionally, FMT upregulated fecal butyric acid levels by 46.7%, and significantly increased propionic acid and isobutyric acid levels by 21.3% and 65.0%, respectively, while no significant changes were seen in other SCFA like caproic, acetic, valeric, or isovaleric acid (36). Levels of propionic acid and butyric acid were positively correlated with BMS scores. Inverse relationships were found between the content of butyric acid and intestinal permeability and the association between isobutyric acid and BMS scores / FITC-dextran permeability was not statistically significant (36).

### Gut microbiota dysbiosis

We identified two human studies (29, 30) and one animal study (28) which explored the role of gut dysbiosis and recovery outcomes following injury. Human studies compared either men with SCI or both men and women with SCI to individuals without SCI, while the animal study used a female C57BL/6 mouse model with an injury at the Th9 level.

A small case–control study examined the association between gut microbiome dysbiosis and biomarkers of inflammation and oxidative stress (30). Men with traumatic cervical SCI exhibited decreased abundances of gut taxa involved in the metabolism of carbohydrates, amino acids, nucleotides, and lipids, as well as reduced transcription and translation activity compared to healthy male individuals (30) At the genus level, *Faecalibacterium, Megamonas, Prevotella_9, Lactobacillus, and Dialister* were significantly less abundant. Meanwhile, *Bacteroides and Blautia* were significantly more abundant in the men with SCI compared to those without the injury. Decreased *Faecalibacterium* correlated with increased triglycerides, Low-Density-Lipoprotein (LDL), and Apolipoprotein B levels, which could harm lipid metabolism. Increased *Bacteroides* were negatively correlated with Apolipoprotein A1, which could be harmful in immunosuppressed individuals with SCI. In contrast, Blautia, which produces SCFA, was positively correlated with uric acid and High-Density Lipoprotein (HDL), indicating that increased levels of Blautia may support improved lipid metabolism in the SCI group (30). Another study compared men and women with thoracic SCI and healthy controls without SCI and associated gut dysbiosis in SCI with changes in serum metabolite profiles and clinical parameters such as injury duration and neurological grade (29). The *UBA1819* abundance was positively correlated with uridine, Lachnospiraceae correlated with hypoxanthine, Blautia with Phosphatidylcholine (18:2/0:0), and Akkermansia with kojic acid. Meanwhile, the abundance of Akkermansia was significantly and negatively correlated with that of 5-{8-(1-(2,4-dihydroxyphenyl)−3-(3,4-dihydroxyphenyl)−2-hydroxypropyl]−3,5,7-trih-ydroxy-3,4-dihydro-2H-1-benzopyran-2-yl} – 2-hydroxyphenyl) ioxidanesulfonic acid in serum. UBA1819 belongs to the family *Ruminococcaceae,* which is SCFA-producing*.* UBA1819 positively correlated with uridine (29), a precursor of pyrimidine nucleotides essential for RNA synthesis and various metabolic processes. Research has shown that uridine can decrease inflammation and oxidative stress, reduce cytotoxicity, and enhance neurophysiological functions. Additionally, it promotes tissue regeneration in mammals and stimulates stem cell activity. These diverse functions highlight uridine's importance in cellular health and regenerative processes. Antibiotic-induced dysbiosis prior to injury in mice resulted in significantly impaired spontaneous locomotor recovery, worsened lesion pathology, and increased intraspinal inflammation compared to controls (28). Spinal cord samples from these mice showed 30% larger total lesion volumes and 25% less spared white matter at the injury epicenter. Notably, these adverse outcomes were substantially ameliorated by initiating commercial probiotic treatment immediately after injury (28).

### Other interventions

We identified three animal studies that linked microbiome modulation to functional recovery in female C57BL/6 mice with Th10 contusion injuries using interventions such as melatonin (35), and MOX (33), and in female BALB/cJRj mice with Th8 spinal cord hemisection treated with rIL-13 (34). Additionally, two studies demonstrated that dietary interventions altering the gut microbiome were associated with improved recovery outcomes in male Sprague–Dawley rats with C5 spinal cord clamping and male Long-Evans rats with Th10 contusion injuries (31, 32).

Melatonin intervention improved motor function and protected against injury-induced gut leakiness and accelerated gastrointestinal transit time in mice (35). There was also a significant increase in the relative abundance of Lactobacillus, belonging to the order Lactobacillales, in SCI mice. Significant changes in taxa were accompanied by an opposite change in minor taxa, including Lachnospiraceae_NK4A136_group and unclassified_f_Lachnospiraceae. The motor scores were positively correlated with the relative abundance of Lactobacillales and Lactobacillus. In contrast, there was an inverse association between motor scores and the relative abundance of Clostridiales. The relative abundances of Lactobacillus and Lactobacillales negatively correlated with gut permeability. The relative abundances of Lactobacillales negatively correlated with Monocyte chemoattractant protein (MCP-1) expression levels. The association between MCP-1 expression levels and the relative abundance of Lactobacillus was not statistically significant (35). The MOX treatment exhibited a greater improvement in locomotor activity, as indicated by higher BMS scores (33). Additionally, the BMS scores were negatively correlated with the abundance of Clostridiales and positively correlated with Lactobacillales levels, suggesting that changes in gut microbiota composition may be linked to recovery outcomes following SCI (33) Similarly, rIL-13 significantly improved motor function 28 days post-injury compared to the vehicle group (34). Regarding gut microbiota composition, Clostridiales vadin BB60 was positively correlated with functional recovery after SCI, regardless of treatment (34). In the rIL-13-treated group, Clostridiales vadin BB60 and Acetitomaculum increased, while *Anaeroplasma*, *Ruminiclostridium_6*, and *Ruminococcaceae UCG-013* and *UCG-010* decreased (34).

Male Sprague–Dawley rats with C5 spinal cord clampingIntermittent fasting (specifically every other day fasting, or EODF) was able to modulate gut microbiota, which was closely associated with neuroprotective-related differentially expressed genes (DEGs) in SCI rats during the acute and subacute phases of injury (32). The transcriptome expression profile revealed that microbial richness under EODF, as indicated by the Chao 1 and ACE indices, was significantly reduced in the EODF-SCI group compared to the ad libitum (AL)-SCI group (32). Additionally, EODF intervention lowered the Simpson index, indicating reduced gut microbiota alpha diversity at multiple time points. At the phylum level, the most abundant taxa in the EODF-SCI, AL-SCI, and AL-SHAM groups were *Firmicutes and Bacteroidetes*. Notably, the EODF-SCI group showed significant increases in anti-inflammatory genera such as *Lactobacillus, and Lachnospiraceae* compared to the AL-SCI group. On day 14 post-injury, *Prevotella1*, *Prevotella9*, and *Lachnospiraceae UCG-008* were more abundant in the EODF-SCI group, while *Bacteroides* levels were significantly decreased (32). Furthermore, Catenibacterium, Lachnospiraceae UCG-008, Odoribacter, Ruminococcaceae UCG-005, Ruminococcaceae UCG-003, Ruminococcus1, and [Eubacterium] coprostanoligenes group were found to have positive correlations with genes associated with anti-inflammatory responses or were negatively correlated with pro-inflammatory genes, suggesting potential neuroprotective effects (32). In another study rats were fed a high  – and low-fat diet (31). There was a significantly higher abundance of Bacteroidales and a lower abundance of *Clostridiales, Feferibacterales, Thermodesulfovibrionales, Xanthomonadales, Rickettsiales, and Myxococcales* in high-fat  – vs. low-fat-fed rats (31). *Clostridiales, Lachnospiraceae and Ruminococcaceae* are linked to higher SCFA production and favorable metabolic outcomes. Thus, a decrease in their abundance with high-fat diet could be disadvantageous to metabolic health in SCI (37).

## Discussion

Neurotrauma, including traumatic brain injury and SCI, alters gut microbial composition and abundance, leading to dysbiosis (5, 10, 38). SCI has been associated with reduced beneficial butyrate-producing bacteria and an increase in potentially pathogenic and pro-inflammatory bacterial species (5). SCFA levels in persons with SCI were significantly decreased compared with individuals without SCI, and there was a clear association between injury severity and duration and SCFA levels (25). SCFAs have beneficial properties via activating immune, vagal, endocrine, and humoral pathways (39) and could be important for the prevention of secondary health complications in individuals aging with SCI.

**Figure 2.**
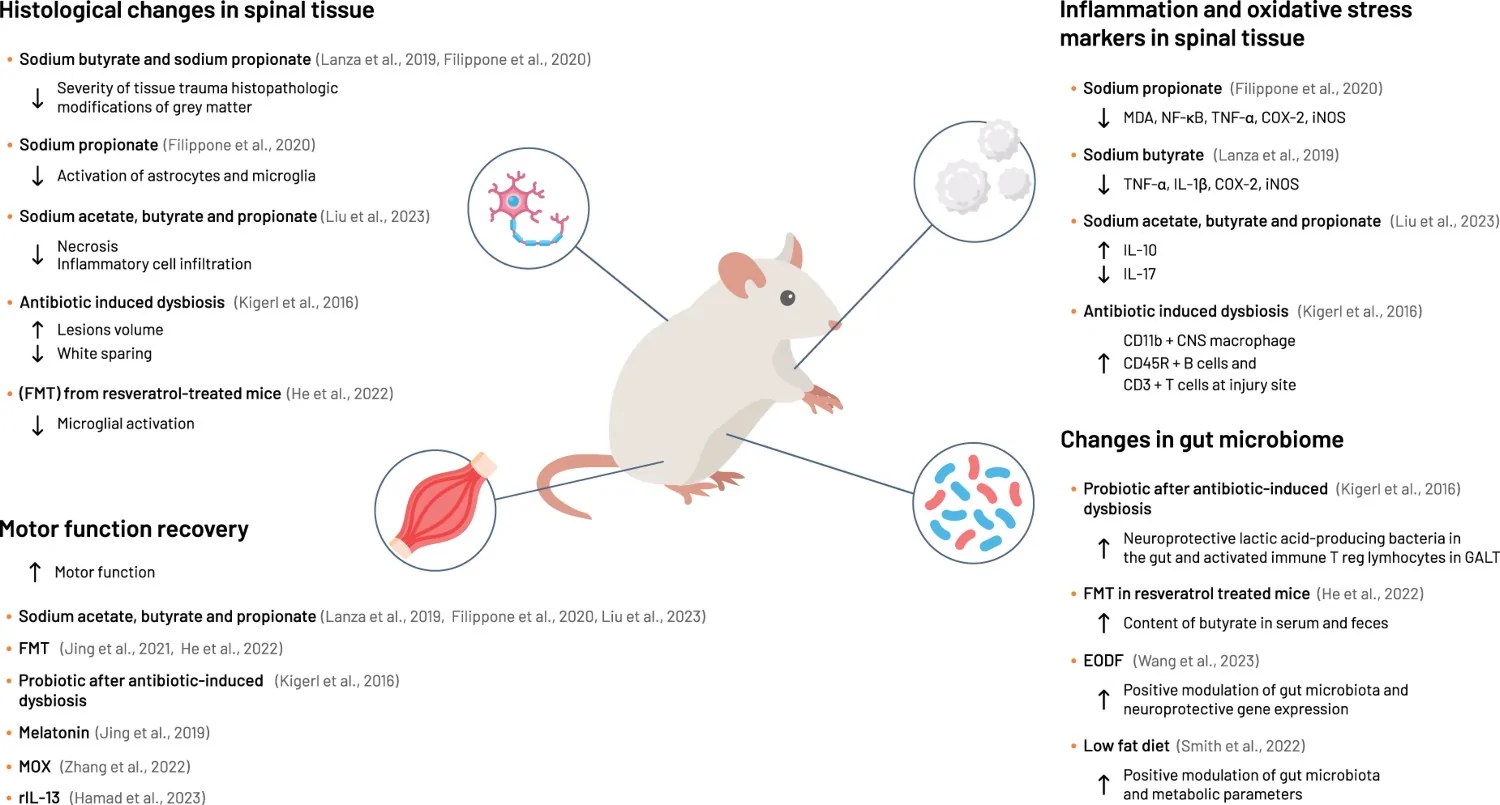
Summary of findings from animal-based models of SCI.

Evidence from animal studies consistently linked SCFA interventions with improved motor function, as all nine studies assessing this outcome reported beneficial effects (Figure 2). Additionally, SCFAs were associated with reduced tissue damage in four out of five studies and with favorable changes in inflammatory and oxidative stress markers in three out of four studies, across diverse SCI models. In line, interventions that induced restoration of gut dysbiosis in SCI (*i.e.* FMT from resveratrol-treated mice or healthy mice and intermittent fasting and interventions such as melatonin, MOX and rIL-13) yield an increase in abundance of SCFA-producing bacteria which were linked with improved inflammation markers in injured animals. Among the two human studies included in this systematic review, associations were found between gut microbiota at the genus and taxa levels and metabolic markers in individuals with SCI. However, the neuroprotective effects of SCFAs have only been examined in animal models. Given species differences, future clinical investigations are needed to confirm the effects of SCFAs’ in humans with SCI.

### Limitations of current review and underlying evidence

We identified only two human studies that explored the association between gut microbiome dysbiosis and biomarkers of inflammation and oxidative stress in men (30) and both men and women with SCI (29). However, neither study measured SCFA levels in blood or stool. Both were subject to selection bias and had notable limitations in their analytic approaches and outcome reporting. Importantly, neither assessed the effectiveness of SCFA-based interventions on neurological or functional recovery.

While findings from animal studies were consistent, none fully met the methodological quality standards defined by SYRCLE’s risk of bias tool. Key limitations included variability in dosing regimens, intervention types and durations, and timing of outcome assessments, factors that can influence the generalizability of results. Only three experimental studies directly evaluated the impact of SCFA interventions on neurological and functional outcomes. Due to this limited evidence base, we also included studies that indirectly explored SCFA-related pathways, such as those involving fecal microbiota transplantation or interventions aimed at increasing the abundance of SCFA-producing bacteria, and results were in line with the main findings on SCFA benefits. Further, the contusion model used in 50% of the included studies, was the most commonly applied animal model of SCI. Although widely used in the literature, contusion injuries are typically induced using transient mechanical forces which can result in a ‘weight bounce’ effect. This effect can lead to inconsistent injury severity and poorly controlled impact duration, undermining reproducibility of the findings.

Moreover, translating findings from animal studies to human patients with SCI remains challenging due to the variability in SCI animal models. Factors such as genetic, immune, and neurophysiological differences – which are specific to species, strain, and sex – can significantly influence the comparability and relevance of findings across studies. Our review revealed a predominance of rodent models, particularly mouse models, with a smaller representation of rats, even though rats, due to their larger size, are easier for surgical manipulations. In both species, strain selection is an important factor to consider, as morphological, sensory, and motor differences have been observed between commonly used strains. For instance, different rat strains can influence the development of SCI and exhibit substrain differences, as nicely illustrated in the case of Sprague–Dawley and Long Evans rats, which are both commonly used in the SCI preclinical field. Sprague–Dawley rats tend to exhibit lower anxiety responses, which may affect behavioral assays, while Long Evans rats are known for greater cognitive resilience post-injury (40). These behavioral differences could lead to varied interpretations of recovery metrics across studies.

Our systematic review found that inbred strains, such as C57BL/6, BALB/cJRj, and C47NL76 mice, are frequently used in SCI research due to their genetic homogeneity, which helps control for genetic variability in immunological and pathological responses (41). However, responses to treatments in genetically homogeneous mice may not fully capture the complexity of human disease, where genetic diversity plays a crucial role in disease progression and treatment outcomes. This could bias the interpretation of the true efficacy of a therapeutic agent when translating results to human patient cohorts. Historically, outbred strains have been promoted to increase genetic variability, thereby enhancing generalizability and potential translational validity. For instance, outbred strains have been shown to exhibit distinct immune responses compared to inbred strains (42). It is also noteworthy that we did not identify any studies using diverse outbred strains or F1 hybrids, which have been suggested as models that could improve translational relevance in SCI research.

Although emerging evidence suggests that the central nervous system responds differently to injury in males and females, and that sex-specific differences in pathophysiological processes exist, preclinical SCI research has still largely focused on female subjects. This is also shown by our study, where 63.6% of animal models of SCI were conducted using female mice. In contrast, clinical studies are predominantly male-dominated, with over 80% of studies including men only or consisting largely of male participants (43, 44).

The rationale for predominantly using female models in SCI research often stems from practical considerations, such as the higher survival rates of female subjects compared to their male conspecifics. Additionally, researchers often select female animals because they can be housed together in the same cages and pooled at later ages without the risk of injuries associated with male aggression. However, this practice presents significant challenges when evaluating the translational potential of preclinical research on supplements or interventions. Both the brain and spinal cord are capable of undergoing morphological changes throughout life which are mainly mediated by gonadal steroids. The neuroprotective effects of both endogenous and exogenous estradiol and progesterone have been linked with improved recovery post-injury, as demonstrated by numerous preclinical and clinical studies. Additionally, it has been suggested that androgens (testosterone and dehydroepiandrosterone, DHEA) may exert sex-specific effects in SCI. Furthermore, inflammatory responses to SCI are sex-dependent, both in terms of cellular recruitment and phenotype. All of this highlights the limitations of relying primarily on female animal models when translating findings to male-dominated human clinical studies. Therefore, future studies should include both sexes, which will further clarify our understanding of sex-specific mechanisms that could have crucial implications for the development of targeted interventions and therapeutic analgesic strategies in SCI patients. Also, it is noteworthy that although epidemiological evidence suggests that women with SCI are typically middle-aged, SCI models often focus on ovary-intact (or ovariectomized) young adult females. Therefore, even when both sexes are included, age and hormonal status should be important factors to consider in the modeling SCI. All of this information may be crucial for formulating new therapeutic strategies for human SCI patients.

## Conclusions

Animal studies consistently demonstrate that SCFAs enhance motor function, reduce tissue damage, and mitigate inflammatory and oxidative stress in SCI models. Interventions aimed at restoring gut microbiota balance, such as FMT and dietary modifications, show significant promise for improving SCI recovery. The consistent findings regarding the beneficial effects of SCFAs across animal models suggest a high degree of reproducibility, underscoring their generalizability and crucial importance for the translational validity of these results. However, despite these promising neuroprotective effects observed in preclinical models, clinical validation is still required to determine the therapeutic potential of SCFAs in human SCI patients.

Limited evidence from human studies linking gut dysbiosis and altered metabolic profiles underscores the relevance of SCFAs in SCI recovery, yet none of the identified studies directly studied the effectiveness of SCFA in neurological or functional recovery post-injury. To fully establish the therapeutic potential of SCFA-based interventions, future research must address several key areas. Randomized controlled trials are urgently needed to evaluate the safety and efficacy of SCFA supplementation in both acute and chronic SCI populations. Concurrently, mechanistic studies are essential to elucidate SCFA interactions with neural repair processes in human tissue. Additionally, the development of targeted delivery systems to enhance SCFA bioavailability, along with investigations into combination therapies that integrate SCFAs with other promising interventions, is critical. These research efforts are pivotal for establishing evidence-based guidelines for the clinical application of SCFA-based therapies in SCI rehabilitation, representing an exciting and innovative frontier in neurorehabilitation research.

## Supplementary Material

Supplemental Material
