# Supplementary material for: The role of short-chain fatty acids in spinal cord injury: A systematic review of human and animal evidence
Source: J Spinal Cord Med. 2026 Feb 19;49(5):927–40. doi: 10.1080/10790268.2025.2607831 (PMC13591990; doi:10.1080/10790268.2025.2607831)
Supplement: Supplementary file 1 [file YSCM_A_2607831_SM7137.docx]

**Supplemental Table 1.** Detailed characteristics of included studies

| **Human studies** | | | | | | | |
| --- | --- | --- | --- | --- | --- | --- | --- |
| **Lead author, publication year** | **Country** | **Study design** | **Study population** | **Injury characteristics/**  **SCI model** | **Exposure/Intervention** | **Comparison groups** | Outcome measures |
| **Kong et al. 2023** | China | Case-control study | 11 individuals with cervical or thoracic SCI (6 male, 5 female)) and 10 healthy controls (6 male, 4 female) | 5 cervical SCI, 6 thoracic SCI, Injury duration 22.81 ± 1.15 months  ASIA grade 4A, 3C, 4D | Not applicable | SCI vs. healthy controls | Gut Microbiome, Serum metabolite profiles |
| **Zhang et al. 2019** | China | Case-control study | 43 men with chronic traumatic complete SCI and 23 healthy controls | Chronic, cervical and thoracic SCI | Not applicable | SCI vs. healthy men | Microbiome and gastrointestinal symptoms |
| **Animal studies** | | | | | | | |
| **Lead author, publication year** | **Country** | **Study design** | **Study population** | **Injury characteristics/**  **SCI model** | **Exposure/Intervention** | **Comparison groups** | **Outcome measures** |
| **Filippone et al. 2020** | Italy | Interventional study | Male CD1 mice, n=160 | Acute Th6-7 spinal extradural compression injury | Treatment with sodium propionate (10mg/kg, 30mg/kg and 100mg/kg) | Vehicle and sham (only laminectomy) | Motor function, histological changes in spinal tissue, inflammation, and oxidative stress markers |
| **Lanza et al. 2019** | Italy | Interventional study | Male CD1 mice, n=120 | Acute Th6-7 spinal extradural compression injury | Treatment with sodium butyrate (10mg/kg, 30mg/kg and 100mg/kg) | Vehicle and sham (only laminectomy) | Motor function, histological changes in spinal tissue, inflammation, and oxidative stress markers |
| **Liu et al. 2023** | China | Interventional study | Female Spraque-Dawleay Rats, n=65 | Acute Th10 spinal contusion injury | SCFA (acetate sodium, propionate sodium and butyrate sodium) | Water | Motor function, histological changes in spinal tissue, inflammation, and oxidative stress markers |
| **Ning He et al. 2022** | China | Interventional study | Female C57BL/6 mice, n= NR | Acute Th10 spinal hemisection injury | Resveratrol Intervention  Intra-gastrically at 200mg/kg/day for 14 days  – SCI + vehicle Group: same method  Fecal microbiota transplantation (FMT)  – fecal pellets from the SCI+Veh and the SCI+Res. group were used to produce a fecal filtrate which was administered intragastrically (200microl) for 7 days to SCI mice  -SCI+FV (recipient)  -SCI+FR (recipient) | Sham group: only laminectomy, no vehicle  Fecal microbiota transplantation (FMT)  control was the SCI+FV group | Motor function, Butyrate concentration in feces, Gut microbiome, SCFA an BCFAs in feces and blood serum, spinal cord and colon tissue samples |
| **Jing, Y et al. 2021** | China | Interventional study | Adult female C57BL/6N mice, n=120 | Acute Th10 spinal contusion Injury | Fecal microbiota transplantation (FMT)^1^ | Vehicle-treated SCI mice | Motor function, spinal cord and colon tissue samples, metabolic parameters (weight, respiratory change quotient), GI transit and permeability, fecal SCFA |
| **Kigerl et al. 2016** | USA | Interventional study | Female C57BL/6 mice, n=NR | Acute Th9 spinal contusion injury | Probiotic after antibiotic-induced dysbiosis | Vehicle-treated SCI mice | Motor function, microbiome changes  Gut permeability |
| **Wang et al. 2023** | China | Interventional study | Male Sprague-Dawley rats, n=72 | Acute C5 spinal injury through temporary aneurysm clamp | Intermittent fasting | Every other day fasting (EODF)-Healthy, Control-Healthy, Control-SCI, Control-SHAM, and EODF-SCI | Microbiome changes, spinal cord tissue samples (transcriptome analysis) |
| **Smith et al. 2022** | USA | Interventional study | Male Long Evans rats, n= NR | Acute Th10 spinal contusion jury | High-fat diet (HFD) and low-fat diet (LFD) | Sham (laminectomy) | Body weight, Microbiome changes |
| **Hamad et al. 2023** | Belgium | Interventional study | Female wild-type (WT) BALB/cJRj mice, n=20 | Acute Th8 spinal hemisection injury | Recombinant IL-13 (rIL-13) | Vehicle | Motor function, microbiome changes |
| **Z. Zhang et al. 2022** | China | Interventional study | Female C47BL76 mice, n=32 | Acute Th10 spinal contusion injury | Moxibustion (MOX) | Vehicle | Motor function, Microbiome changes |
| **Jing et al. 2019** | China | Interventional study | Female C57BL/6 mice, n=64 | Acute Th10 contusion injury | Melatonin treatment | Vehicle/sham and vehicle/SCI  Group vs. Sham and SCI Melatonin treatment groups | Motor function, Microbiome changes, Colon tissue samples, GI motility metabolic parameters |

**Search strategy**

**Search date: 19.10.2023**

|  | Before deduplication | After deduplication |
| --- | --- | --- |
| Medline Ovid | 445 |  |
| Web-of-Science | 566 |  |
| Embase.com | 1347 |  |
| Total | 2358 | **1965** |

393 duplicate records have been removed

Medline via Ovid

exp Fatty Acids, Volatile/ or (short chain fatty acid* or short-chain fatty acid* or SCFA* or Acetates or acetate or Propionates or propionate or Butyrates or butyrate).ab,ti.

OR

"Gastrointestinal Microbiome"/ or "Bacterial Load"/ or "Dysbiosis"/ or exp "Bacteroidaceae"/ or "Microbiota"/ or "Microbial Consortia"/ or dysbios*.mp. or dys-symbios*.mp. or dysbacterios*.mp. or disbacterios*.mp. or ((enteric* or alimentary-tract* or bowel or intestin* or gastro-intestin* or gastrointestin* or gut or colon* or fecal or feces) adj3 (flora or microflora* or micro-flora* or bacteri* or microorganism* or micro-organism* or microbi*)).ab,ti.

AND

exp Spinal Cord Injuries/ or exp Spinal Cord Ischemia/ or exp Paraplegia/ or Quadriplegia/ or Spinal Dysraphism/ or (((spine or spinal) adj3 (injur* or trauma* or 3ensit*)) or (spinal cord adj3 (disease* or contusion* or laceration* or transection* or lesion* or trauma* or ischemi* or ischaemi*)) or (3ensitive3* adj3 (trauma* or post-trauma* or posttrauma*)) or ((spine or spinal or vertebra*) adj3 (fracture* or trauma* or injur* or damage* or wound*)) or central cord injury syndrome* or central cord syndrome* or central spinal cord syndrome* or cauda equine syndrome* or anterior cord syndrome* or conus medullaris syndrome* or Brown Sequard or paraplegi* or quadriplegi* or tetraplegi*).ab,ti.

Web of Science

TS=((((spine or spinal) NEAR/3 (injur* or trauma* or damag*)) OR (("spinal cord" OR "spine cord" OR "cervical cord") NEAR/3 (injur* or trauma* or damag* or disease* or contusion* or laceration* or transection* or transsection* or section* or lesion* or trauma* or ischemi* or ischaemi* or syndrome*)) OR (myelopath* NEAR/3 (trauma* or post-trauma* or posttrauma* or ischemi* or ischaemi*)) OR ((spine or spinal or vertebra*) NEAR/3 (fracture* or trauma* or injur* or damag* or wound*)) OR "central cord injury syndrome*" OR "central cord syndrome*" OR "central spinal cord syndrome*" OR "cauda equine syndrome*" OR "anterior cord syndrome*" OR "conus medullaris syndrome*" OR (transverse NEAR/3 lesion*) OR "Brown Sequard*" OR paraplegi* OR quadriplegi* OR tetraplegi*)) AND TS=(scfa* OR short chain fatty acid* OR ((fatty** NEAR/3 (short OR chain)) OR short-chain fatty acid* OR acetate OR propionate OR Propionic OR Butyric OR Butyrate OR valeric OR Valerate OR Formic OR formate ) OR microbiome or microbiota OR dysbios* OR dys-symbios* OR dysbacterios* OR disbacterios* OR ((enteric* OR alimentary-tract* OR bowel OR intestin* OR gastro-intestin* OR gastrointestin* OR gut OR colon* OR fecal OR feces) NEAR/2 (flora OR microflora* OR micro-flora* OR bacteri* OR microorganism* OR micro-organism* OR microbi*)))

Embase

'short chain fatty acid'/de OR 'acetate'/exp OR acetate OR 'propionate'/exp OR propionate OR 'butyrate'/exp OR butyrate

OR

'dysbiosis'/de OR 'bacteroidaceae'/exp OR 'microflora'/de OR 'microbiome'/exp OR 'microbial consortium'/de OR 'microbial diversity'/de OR dysbios* OR 'dys symbios*' OR dysbacterios* OR disbacterios* OR ((enteric* OR 'alimentary tract*' OR bowel OR intestin* OR 'gastro intestin*' OR gastrointestin* OR gut OR colon* OR fecal OR feces) NEAR/3 (flora OR microflora* OR 'micro flora*' OR bacteri* OR microorganism* OR 'micro organism*' OR microbi*))

AND

'spinal cord injury'/exp OR 'cervical spine injury'/de OR 'spinal cord ischemia'/de OR 'paraplegia'/de OR 'spastic paraplegia'/de OR 'quadriplegia'/de OR 'spinal dysraphism'/de OR 'neurogenic bowel'/de OR ('injury'/exp AND 'spinal cord'/exp) OR (((spine OR spinal) NEAR/3 (injur* OR trauma* OR damag*)):ab,ti) OR (('spinal cord' NEAR/3 (disease* OR disorder* OR contusion* OR laceration* OR transection* OR lesion* OR trauma* OR 'post trauma*' OR ischemi* OR ischaemi*)):ab,ti) OR ((myelopath* NEAR/3 (trauma* OR 'post trauma*' OR posttrauma*)):ab,ti) OR (((spine OR spinal OR vertebra*) NEAR/3 (fracture* OR trauma* OR injur* OR damage* OR wound*)):ab,ti) OR 'central cord injury syndrome*':ab,ti OR 'central cord syndrome*':ab,ti OR 'central spinal cord syndrome*':ab,ti OR 'cauda equine syndrome*':ab,ti OR 'anterior cord syndrome*':ab,ti OR 'conus medullaris syndrome*':ab,ti OR 'brown sequard':ab,ti OR paraplegi*:ab,ti OR quadriplegi*:ab,ti OR tetraplegi*:ab,ti OR wheelchair*:ab,ti OR paralympi*:ab,ti OR 'para athlet*':ab,ti OR parathlet*:ab,ti OR 'para sport*':ab,ti OR (((neurogenic OR neuropathic) NEAR/2 (bowel* OR bladder*)):ab,ti) OR ((('lower limb*' OR 'lower extremit*') NEAR/3 paralys*):ab,ti)
